# Role of Intracranial Aneurysm Location in Determining Optimal Management: A Systematic Review and Meta-Analysis of Randomized Controlled Trials

**DOI:** 10.7759/cureus.96040

**Published:** 2025-11-03

**Authors:** Muhammad Irfan, Masum Rahman, Anshum Patel, Muhammad Sajjad, Zermina Tanvir, Aishwarya S Shikhare

**Affiliations:** 1 Neurosurgery, Nishtar Medical University, Multan, PAK; 2 Neurosurgery, Mayo Clinic, Rochester, USA; 3 Medicine and Surgery, Narendra Modi Medical College, Ahmedabad, IND; 4 Medicine, Topiwala National Medical College and Bai Yamunabai Laxman (BYL) Nair Charitable Hospital, Mumbai, IND

**Keywords:** aneurysm coiling, aneurysm location, functional outcome, mortality, surgical clipping, systematic review and meta-analysis

## Abstract

This meta-analysis aims to evaluate the influence of aneurysm location on optimal management strategies, specifically comparing clipping and coiling.

We conducted a systematic review and meta-analysis of randomized controlled trials (RCTs) collected from PubMed, Scopus, and Google Scholar up to December 2024. The mixed-effects model reported a dichotomous outcome using the risk ratio (RR) with a 95% confidence interval (CI). Subgroup analysis was performed, and statistical heterogeneity was evaluated using I² statistics. Bias was assessed using funnel plots and Egger's test. The primary outcome included the mortality rate among clipping and coiling treatments, and the secondary endpoint was a favorable functional outcome at 1-5 years of follow-up.

We included eight RCTs, totaling 8199 patients, and the qualitative systematic review analysis showed that most aneurysms are located in the anterior circulation, with a predominantly female distribution. Clipping has been beneficial for middle cerebral artery (MCA) and anterior communicating artery (AComA) aneurysms. Meanwhile, coiling for the posterior circulation carries a higher risk of rupture and rebleeding. In quantitative meta-analysis, coiling showed a significant reduction of mortality as compared to clipping, with a pooled RR (RR = 0.84; 95% CI: 0.75-0.95; P = 0.05; I² = 48%). Favorable functional outcome was seen as more significant in coiling than clipping (RR = 0.9; 95% CI: 0.80-1.01; I² = 25.7%; τ² = 0.0052; P = 0.224) using the random-effects model. The subgroup analysis for AComA aneurysms indicated that coiling seemed to lead to safer results (RR = 0.88; 95% CI: 0.75-1.04), while for MCA aneurysms, clipping appeared to be better (RR = 1.72; 95% CI: 0.78-3.77). In the case of internal carotid artery (ICA) aneurysm, coiling has been associated significantly with a lower rate of mortality and dependency (RR = 0.60; 95% CI: 0.49-0.75), and the posterior circulation did not reveal any association of outcome with either treatment (RR = 0.97; 95% CI: 0.11-8.86).

The evidence suggests that coiling has been beneficial in terms of overall mortality reduction and better functional outcomes. However, clipping has shown effectiveness for MCA aneurysms, and coiling has proven to be a slightly safer modality for AComA and a significantly preferable modality for ICA aneurysms. No significant association of outcome was found among the posterior circulation, clipping, or coiling.

## Introduction and background

Aneurysmal subarachnoid hemorrhage (aSAH) is a life-threatening neurosurgical emergency due to the rupture of an intracranial aneurysm, having an overall global incidence of nine per 100,000 person-years, which comprises approximately 5% of all strokes [1,2] and could cause population-based mortality rates ranging from 8% to 67%, which is higher when associated with rebleeding and long-term neurological morbidity. Its incidence increases with age [3,4]. Therefore, urgent aneurysm occlusion is critical to prevent rebleeding. Two primary treatment modalities, like neurosurgical clipping and endovascular coiling, are generally used for aneurysm management; however, various factors such as patient age, aneurysm morphology, preoperative neurological clinical grade, and aneurysm location help in the choice between surgical clipping and coiling [5]. Both treatment modalities have emerged significantly based on aneurysm locations because of multiple large-scale trials and cohort studies, such as a study that has shown that endovascular treatment is feasible in 95% of patients using the latest advances like three-dimensional imaging and intervention material [6,7], but there is still some controversy in the selection of favorable intervention based on specific locations. Anatomical location plays a key role in determining its choice due to its crucial contribution to procedural feasibility, complication risk, and long-term outcome because there is great variation among different aneurysm sites, such as the anterior communicating artery (AComA), the internal carotid artery (ICA), and the posterior circulation, in terms of their approach, structure, and surrounding vascular structures, affecting procedural risk, technical complexity, and eventually outcomes of treatment. As it has been shown in a trial, middle cerebral artery (MCA) aneurysms are easily accessible surgically and may favor clipping [8,9], whereas the posterior circulation is generally considered more suitable for coiling due to higher surgical risks. While previous literature has compared the efficacy and safety of coiling versus clipping, it mainly focuses on general outcomes without adequately emphasizing the influence of aneurysm location by its stratification. As a result, current guidelines often provide generalized recommendations, leading to a lack of consensus in clinical practice regarding location-specific management strategies [10]. This systematic review and meta-analysis aim to address this gap by evaluating existing randomized controlled trials (RCTs) to determine how aneurysm location impacts the comparative outcomes of coiling and clipping. By stratifying results based on aneurysm sites, we seek to provide evidence-based insights to guide tailored treatment decisions and improve patient-specific care strategies.

## Review

Materials and methods

Study Design and Protocol

This systematic review and meta-analysis rigorously followed the guidelines outlined in the Preferred Reporting Items for Systematic Reviews and Meta-Analyses (PRISMA) [11] and the Cochrane Handbook of Systematic Review and Meta-Analysis.

Literature Search Strategy

Two independent reviewers systematically searched electronic databases, including PubMed, Google Scholar, and Scopus, until December 2024 without any search limits or filters. The search terms used were "intracranial aneurysm", "subarachnoid hemorrhage", "clipping vs. coiling", "aneurysm location", and "RCT". Detailed baseline information and outcomes are provided.

Eligibility Criteria

RCTs meeting the following population, intervention, comparison, and outcome (PICO) criteria were included like populations with intracranial aneurysms, surgical treatment of aneurysms including microsurgical clipping and endovascular coiling, comparison between anterior circulation aneurysms, such as the AComA and MCA, and posterior circulation aneurysms, such as the basilar artery and posterior cerebral artery (PCA), and outcome reporting mortality, treatment failure, and functional outcome, such as the modified Rankin Scale (mRS = 0-2), and exclusion criteria comprised studies that were not RCTs, animal and preclinical studies, and non-peer-reviewed studies such as abstracts or editorials. After the removal of duplicates, four team members independently screened titles and abstracts of extracted records for eligibility. Full texts of selected studies were retrieved and screened for inclusion in the meta-analysis. Any discrepancies were removed by the other two team members.

Data Extraction

Two team members independently formulated data extraction sheets for the following data: study characteristics collected (authors, publication year, sample size), aneurysm location (anterior circulation, like AComA, ICA, and MCA, or posterior circulation, like basilar artery, PCA, and posterior communicating artery (PComA)), treatment received (clipping vs. coiling), and outcome (primary, such as mortality or treatment failure, and secondary, such as functional independence, e.g., mRS = 0-2).

Risk of Bias and Certainty of Evidence

We estimated the risk of bias in the included studies using the revised Cochrane Collaboration tool for RCTs (Risk of Bias 2 (RoB2)) [12]. Three reviewers individually evaluated studies for selection, performance, reporting, attrition, and overall bias, with disagreements resolved with consensus. Four team members evaluated the bias related to the randomization process, deviations from the intended intervention, missing outcome data, measurement of outcomes, selection of reported results, and overall biases, reaching a consensus on their findings.

Statistical Analysis

The R software (version 4.3) (R Foundation for Statistical Computing, Vienna, Austria) performed the statistical analysis using the meta, metafor, and dmetar packages, along with the RevMan software (The Cochrane Collaboration, London, UK). The effect size calculation was done with a risk ratio (RR) to pool the results with a 95% confidence interval (CI) using a random-effects model when there was significant heterogeneity (I^2^ > 50%) and a fixed-effects model when heterogeneity was not substantial (I^2^ < 50%). The I^2^ was interpreted as no significance for 0-40%, moderate heterogeneity for 30-60%, substantial heterogeneity for 50-90%, and considerable heterogeneity for 75-100%, following the Cochrane Handbook. Subgroup analysis was performed to stratify the aneurysm location, including AComA, MCA, ICA, PComA, PCA, and basilar artery. We performed Egger's test, funnel plot, and trim-and-fill methods to assess the publication bias, along with the Metabias R function.

Results

Study Selection

**Figure 1:**
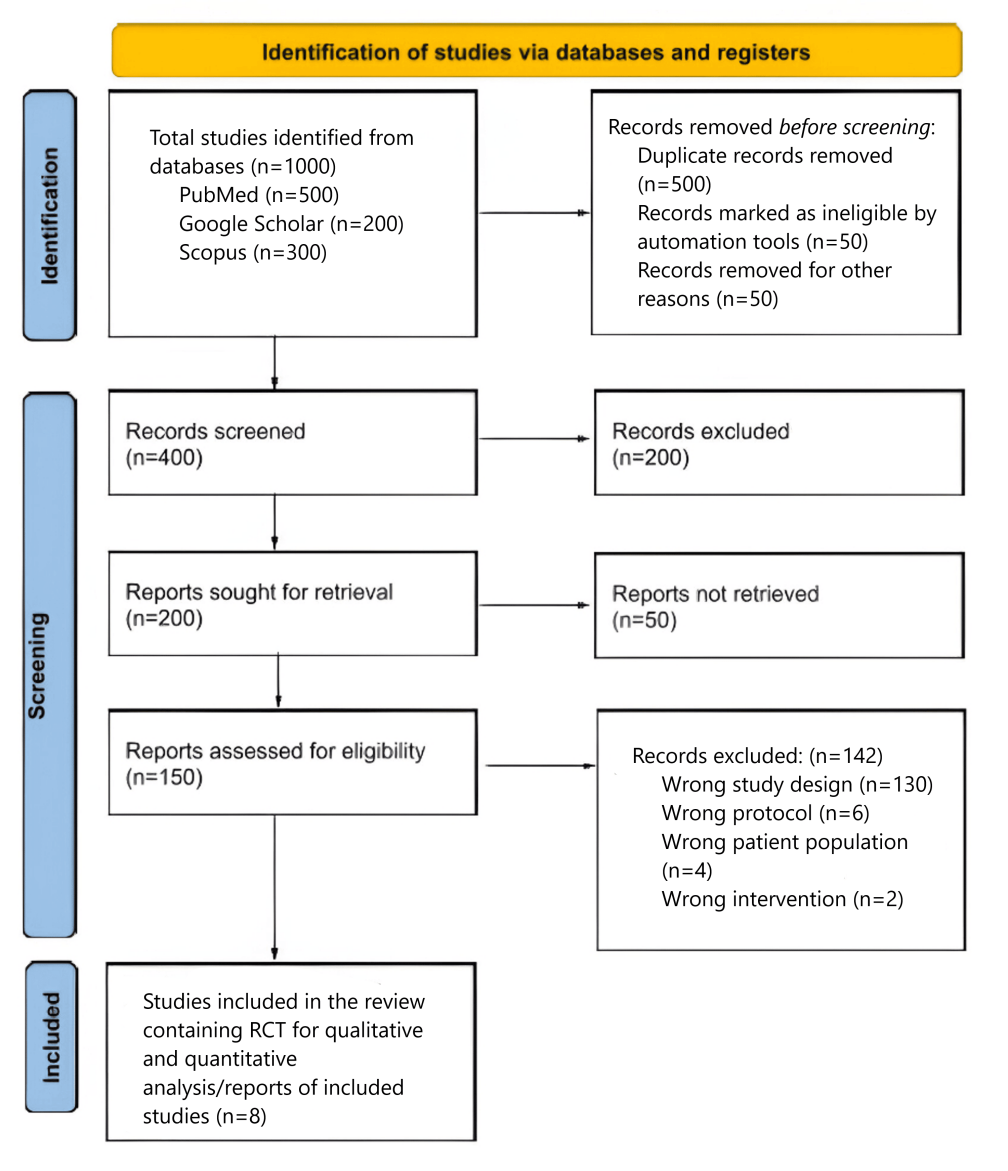
PRISMA 2020 flow diagram showing the search of databases and registers for the current meta-analysis PRISMA: Preferred Reporting Items for Systematic Reviews and Meta-Analyses; RCT: randomized controlled trial

During the search strategy, 1000 articles were selected from the database, and screening was performed based on titles and abstracts. Out of the 1000 articles, 500 duplicates were removed, in addition to 50 marked as ineligible by automation tools and 50 removed for other reasons. With 400 articles left, 200 records that did not fulfill the eligibility criteria were excluded; hence, 200 articles were sought for retrieval, of which 50 articles were not retrieved, leaving only 150 articles assessed for eligibility criteria. Out of the 150 articles, 142 records were excluded, that is, 130 due to the wrong study design, six due to the wrong protocol, four due to the wrong patient population, and two due to the wrong intervention, as described by the PRISMA flow diagram (Figure 1). Eight studies [8,13-19] were included for quantitative and qualitative analysis.

Risk of Bias and Certainty of Evidence

**Figure 2:**
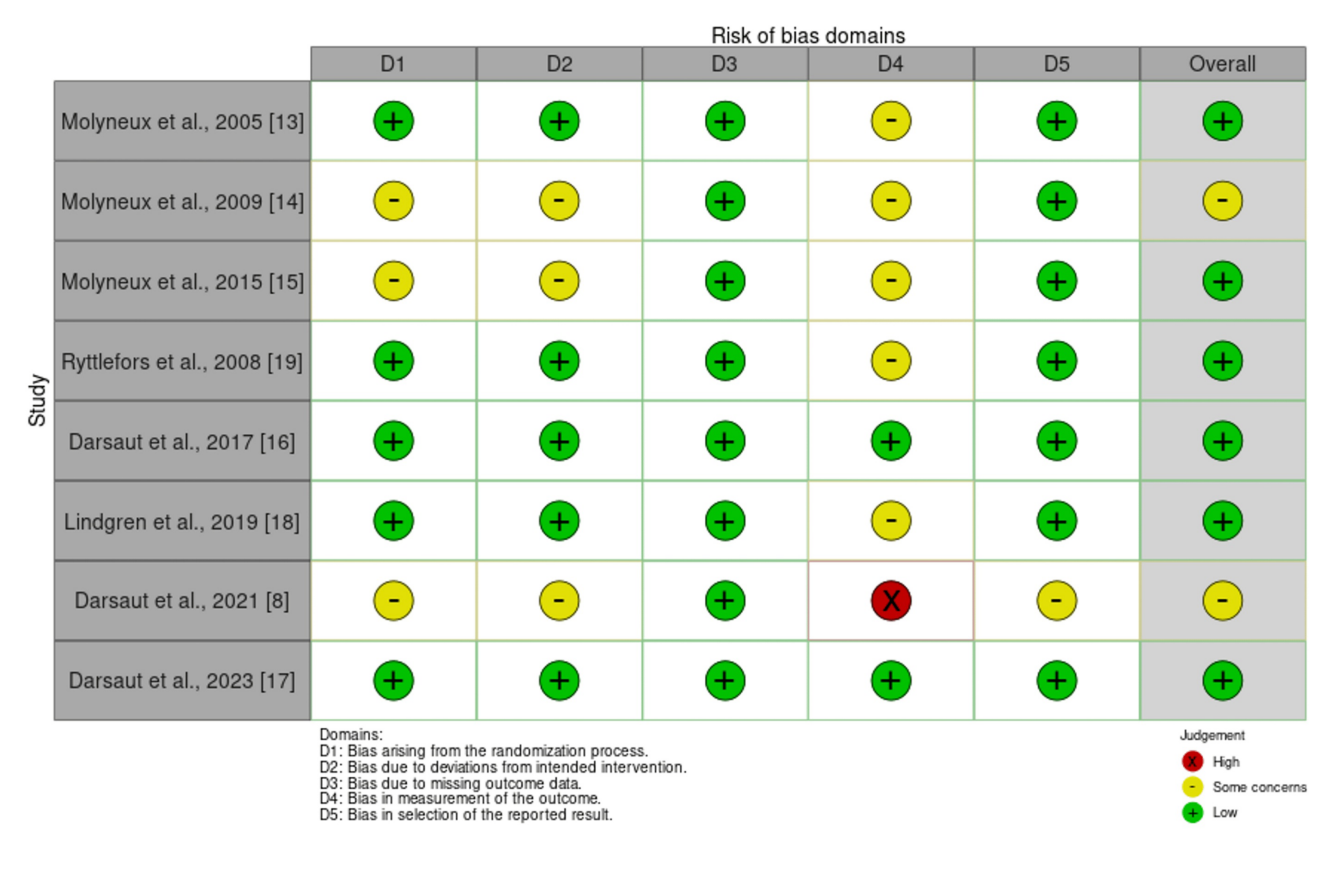
RoB2 tool for the randomized controlled trials included in the meta-analysis RoB2: Risk of Bias 2

**Figure 3:**
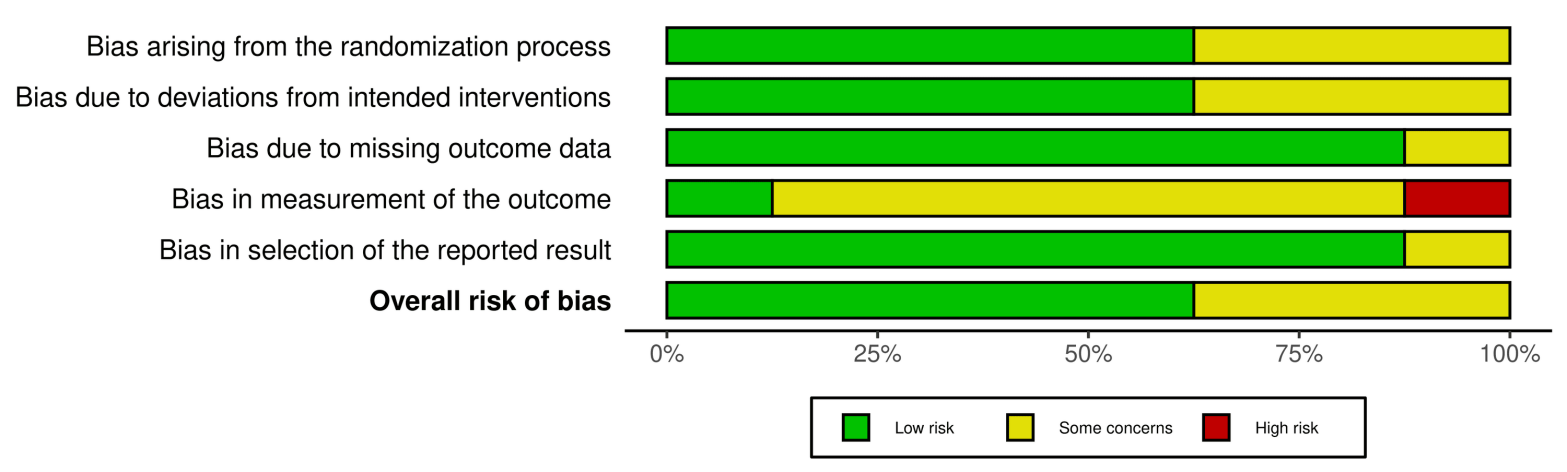
RoB2 risk of bias assessment summary plot for the randomized controlled trials included in the meta-analysis RoB2: Risk of Bias 2

The RoB2 tool showed that Molyneux et al., Darsaut et al., Darsaut et al., Lindgren et al., and Ryttlefors et al. had an overall low risk of bias [13,16-19]. However, Darsaut et al., Molyneux et al., and Molyneux et al. [8,14,15] had some overall concerns because of biases arising from the randomization process, deviations from the intended treatment, and selection of reported results (Figure 2 and Figure 3).

Characteristics of the Included Studies

Only eight RCTs were included, totaling 8199 patients, with 4193 in the coiling group and 4006 in the clipping group. The average follow-up duration was 1-5 years. The detailed information about the included RCTs' summary characteristics and participants' baseline characteristics is reported in Table 1, and the outcome of the included studies' patients is given in Table 2. The mean age of the patients was 56 years, with a gender distribution of more females than males. The 90-95% aneurysm location was in the anterior circulation, and 5-10% were in the posterior circulation, with specific locations such as AComA (48-50%), ICA (23-33%), PComA (3%), anterior inferior cerebellar artery (7%), posterior inferior cerebellar artery (PICA) (1.1%), and MCA (13-14%). The ruptured status was 87.5%, and the non-ruptured status was 12.5%. The aneurysm size was less than 10 mm in most patients. The preoperative neurological status was classified as World Federation of Neurosurgical Societies (WFNS) grades 1-2. The WFNS grading system is a clinical scale used to assess the severity of subarachnoid hemorrhage (SAH) based on a patient's Glasgow Coma Scale (GCS) score and the presence or absence of focal neurological deficits.

**Table 1:** Baseline characteristics of the eight included studies WFNS: World Federation of Neurosurgical Societies; EVT: endovascular therapy; NS: neurosurgical clipping; AComA: anterior communicating artery; MCA: middle cerebral artery; ICA: internal carotid artery; PComA: posterior communicating artery; PICA: posterior inferior cerebellar artery

Authors	Publication year	Sample size	Mean age	Gender distribution (%)	Aneurysm location	Specific location	Aneurysm size	Rupture status	Preoperative neurological status
Molyneux et al., 2005 [13]	2005	Total = 2143; EVT = 1073; NS = 1070	52	Male = 37%; female = 63%	Anterior circulation (95%); posterior circulation (5%)	AComA = 49.8%; MCA = 14.9%; ICA = 32.3%; posterior = 2.7%	90% < 10 mm	Yes	WFNS grade = 1 or 2
Molyneux et al., 2009 [14]	2009	Total = 2087; EVT = 1041; NS = 1046	52	Male = 37%; female = 63%	Anterior circulation (95%); posterior circulation (5%)	AComA = 49.8%; MCA = 14.9%; ICA = 32.3%; posterior = 2.7%	90% < 10 mm	Yes	WFNS grade = 1 or 2
Molyneux et al., 2015 [15]	2015	Total = 1644; EVT = 809; NS = 835	52	Male = 37%; female = 63%	Anterior circulation (95%); posterior circulation (5%)	AComA = 49.8%; MCA = 14.9%; ICA = 32.3%; posterior = 2.7%	90% < 10 mm	Yes	WFNS grade = 1 or 2
Ryttlefors et al., 2008 [19]	2008	Total = 278; EVT = 138; NS = 140	68	Female = 71%; male = 29%	Anterior circulation (90%); posterior circulation (10%)	AComA = 48.9%; ICA = 23.7%; PComA = 12.6%; MCA = 13.3%; basilar artery = 0.4%; PICA = 1.1%	100% < 10 mm	Yes	WFNS grade = 1 or 2
Darsaut et al., 2017 [16]	2017	Total = 134; EVT = 70; NS = 64	57	Female = 62%; male = 38%	Anterior circulation (90.7%); posterior circulation (9.3%)	AComA = 50%; other anterior = 29.6%; posterior = 9.3%	77.9% < 10 mm	Yes	Not mentioned
Lindgren et al., 2019 [18]	2019	Total = 1501; EVT = 852; NS = 649	55	Female = 70%; male = 30%	Anterior circulation (87.4%); posterior circulation (12.6%)	Specific locations not mentioned	85.8% ≤ 10 mm	Yes	WFNS grade = 1 or 2
Darsaut et al., 2021 [8]	2021	Total = 161; EVT = 80; clipping = 81	58	Not specifically mentioned	Anterior circulation (87.4%); posterior circulation (12.6%)	MCA = 14%; other locations not specified	Median = 6 mm	Yes	WFNS grade = 1 or 2; Fisher grade = 0-2
Darsaut et al., 2023 [17]	2023	Total = 280; clipping = 138; coiling = 142	56	Female = 70%; male = 30%	Anterior circulation (97%); posterior circulation (3%)	Aneurysm location not specified	3-25 mm	No	WFNS grade = 1 or 2

**Table 2:** Outcomes of the patients from the eight included studies

Study	Mortality rate	Morbidity risk	Rebleeding or rupture or vasospasm risk	Functional recovery rate
Molyneux et al., 2005 [13]	23.3% = coiling; 30% = clipping	Not mentioned	Higher rebleeding risk = coiling	76.6% = coiling; 30% = clipping at 1 year
Molyneux et al., 2009 [14]	11% = coiling; 14% = clipping at 5 years	Not mentioned	Higher rebleeding risk = coiling	83% = coiling; 82% = clipping at five years
Molyneux et al., 2015 [15]	17% = coiling; 21% = clipping	Not mentioned	Higher rebleeding risk = coiling	82% = coiling; 78% = clipping at 10 years
Ryttlefors et al., 2008 [19]	18.1% = coiling; 21.6% = clipping at 1 year	Higher epilepsy risk = clipping	Higher rebleeding risk = clipping	60.1% = coiling; 56.1% = clipping at 1 year
Darsaut et al., 2017 [16]	3.6% = coiling; 4.2% = clipping at 1 year	Higher epilepsy risk = clipping	Not mentioned	90% = coiling; 76.4% = clipping at 1 year
Lindgren et al., 2019 [18]	6.4% = clipping; 8.2% = coiling at 14-day case fatality	Not mentioned	Not mentioned	32.4% = clipping; 30.2% = coiling at 90 days
Darsaut et al., 2021 [8]	24% = coiling; 7% = clipping at 1 year	Not mentioned	Higher vasospasm = coiling	76% = coiling; 93% = clipping at 1 year
Darsaut et al., 2023 [17]	0.7% = coiling; 0.7% = clipping	Not mentioned	Higher rebleeding risk = clipping	99.6% = clipping; 99.6% = coiling at 1 year

In the qualitative analysis, clipping showed a higher success rate for AComA and MCA aneurysms but increased risk of epilepsy and mortality and a poor functional recovery rate. It has been described in some of the included trials that treatment failures for MCA were more common in the endovascular group, indicating this location is at higher risk for endovascular therapy (EVT), as 24% of deaths were reported in the endovascular group and 7% in the surgical group for MCA aneurysms in the International Subarachnoid Aneurysm Trial-2 (ISAT-2) and Collaborative UnRuptured Endovascular vs. Surgery (CURES) trials. While coiling was found to be beneficial for internal carotid and posterior circulation, it has shown a high risk of rupture and rebleeding. However, there were lower mortality and better functional recovery rates in coiling than in clipping at the one- and five-year follow-up.

Main Meta-Analysis

**Figure 4:**
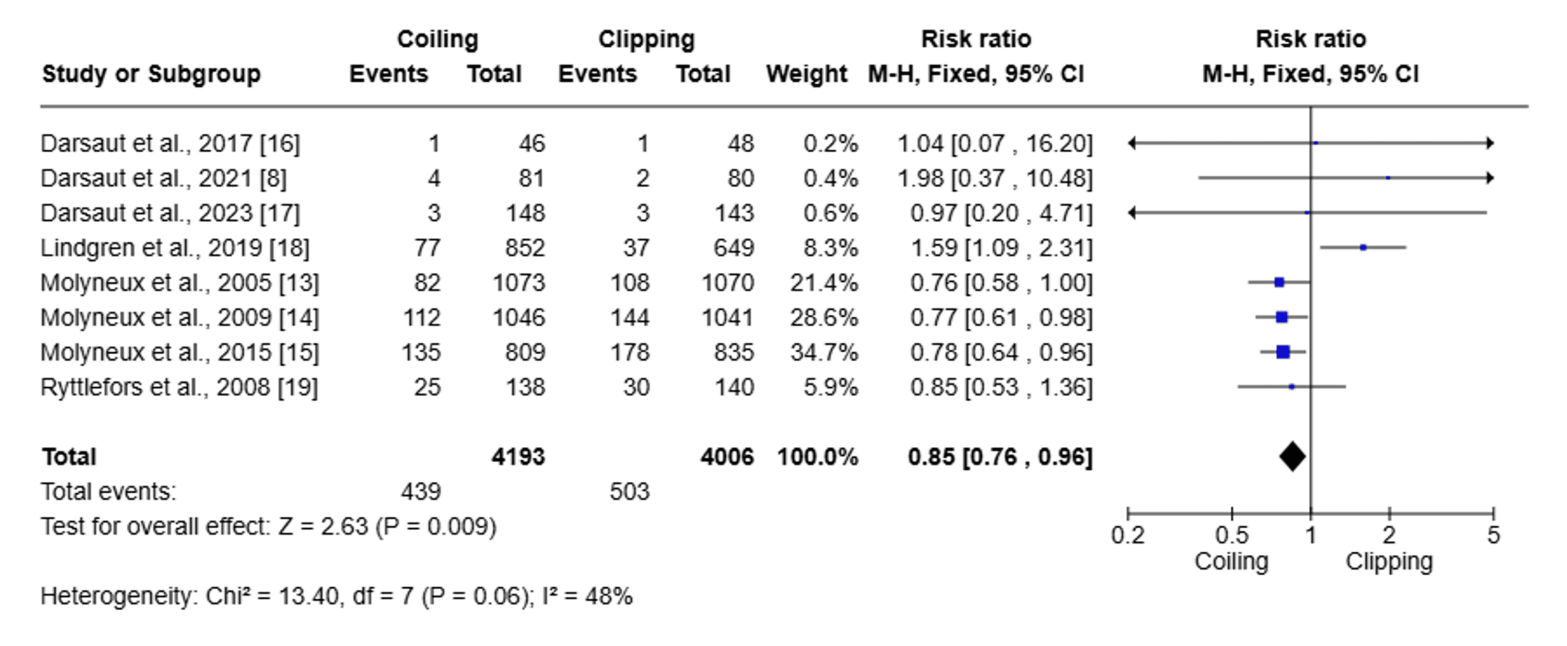
Forest plot for mortality risk associated with clipping and coiling treatment

**Figure 5:**
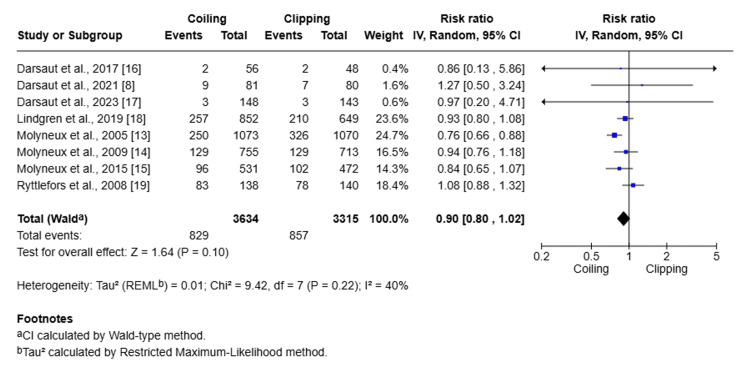
Forest plot for secondary outcome such as functional independence associated with clipping and coiling

Meta-analysis was performed to determine the primary outcome, such as mortality risk, and the secondary outcome, such as improved functional outcome, associated with clipping and coiling treatment (Figure 4 and Figure 5).

Eight studies involving a total of 8199 patients (4193 undergoing coiling and 4006 undergoing clipping) were analyzed to assess the mortality associated with either clipping or coiling, as indicated by the forest plot. The RR of 0.85 (95% CI: 0.76-0.96; P = 0.005) indicates that coiling significantly reduces mortality compared to clipping (I^2^ = 48%); heterogeneity was only modest. Overall, coiling was associated with a 16% reduction in death rate.

A total of eight studies comprising 6949 patients (3634 in the coiling group and 3315 in the clipping group) were included in the meta-analysis for a good functional outcome, defined as an mRS ≤2 at follow-up. The forest plot showed that the pooled RR using the random-effects model was 0.90 (95% CI: 0.80-1.02), favoring coiling over clipping, although the difference did not reach statistical significance (P = 0.0615). It indicates a 10% relative improvement in favorable outcome with coiling compared to clipping, but with CI crossing the zero line having no effect, heterogeneity across studies has low to moderate I^2^ at 25.7%.

*Sensitivity Analysis* 

A leave-one-out sensitivity analysis was performed to evaluate the robustness of the findings, and pooled RR remained largely stable, ranging from 0.86 to 0.95, with no single study significantly altering the overall estimate; however, omitting Molyneux et al. or Ryttlefors et al. mildly shifted the effect toward null, suggesting these studies exerted the greatest influence [13,19]. The Baujat plot showed that Molyneux et al. had the biggest impact on both the differences in results and the overall outcome, followed by Ryttlefors et al., while the other studies had little effect on the differences [13,19].

**Figure 6:**
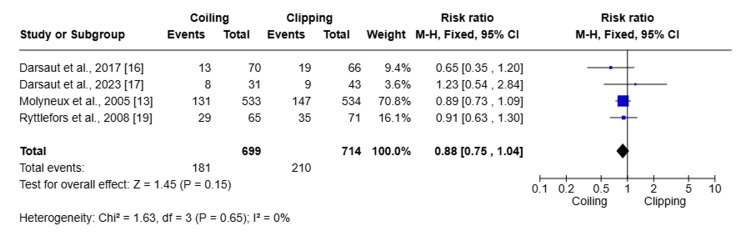
Forest plot showing the subgroup analysis of clipping and coiling for AComA aneurysms AComA: anterior communicating artery

 Subgroup analysis was done for AComA aneurysms comparing clipping and coiling (Figure 6).

A total of 1413 patients were included in four studies comparing the coiling and clipping of AComA aneurysms, and the pooled RR for mortality or dependence favored coiling (RR = 0.88; 95% CI: 0.75-1.04), although this difference was not statistically significant (Z = 1.45; P = 0.15). Heterogeneity was low (I^2^ = 0%), indicating consistency across studies. Overall, the data show a trend toward better outcomes with coiling, but the evidence does not support a statistically significant difference between treatments.

**Figure 7:**
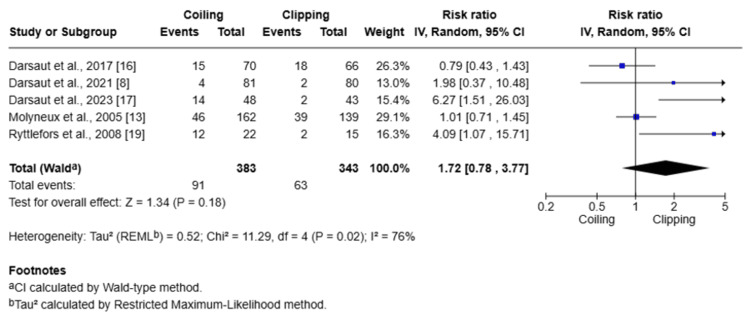
Forest plot showing the subgroup analysis of clipping and coiling for MCA aneurysms MCA: middle cerebral artery

Subgroup analysis was done for MCA aneurysms comparing clipping and coiling (Figure 7).

Five studies involving 726 patients were included in the analysis of MCA aneurysms. The pooled analysis using the random-effects model showed a non-significant trend favoring neurosurgical clipping over coiling. The overall pooled RR is 1.72 (95% CI: 0.78-3.77), with Z = 1.34 and P = 0.18, highlighting a higher but non-significant rate of poor outcomes such as mortality and dependence in the coiling group. Significant heterogeneity was seen (I^2^ = 76%; Chi^2^ = 11.29; P = 0.02), indicating variability of treatment effects across studies. Despite the lack of statistical significance, individual studies such as Darsaut et al. and Ryttlefors et al. demonstrated a significantly higher risk with coiling [17,19].

**Figure 8:**
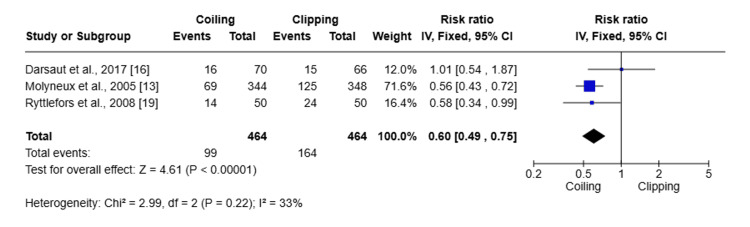
Forest plot showing the subgroup analysis of clipping and coiling for ICA aneurysms ICA: internal carotid artery

Subgroup analysis was done for ICA aneurysms comparing clipping and coiling (Figure 8).

A total of 928 patients were included across three studies assessing ICA aneurysms. The pooled analysis yielded a statistically significant advantage for coiling over clipping (RR = 0.60; 95% CI: 0.49-0.75; Z = 4.62; P < 0.00001), with moderate heterogeneity (I² = 33%). These findings suggest that coiling is associated with significantly lower rates of mortality or dependence in patients with ICA aneurysms and may be the preferred treatment modality in this subgroup.

**Figure 9:**
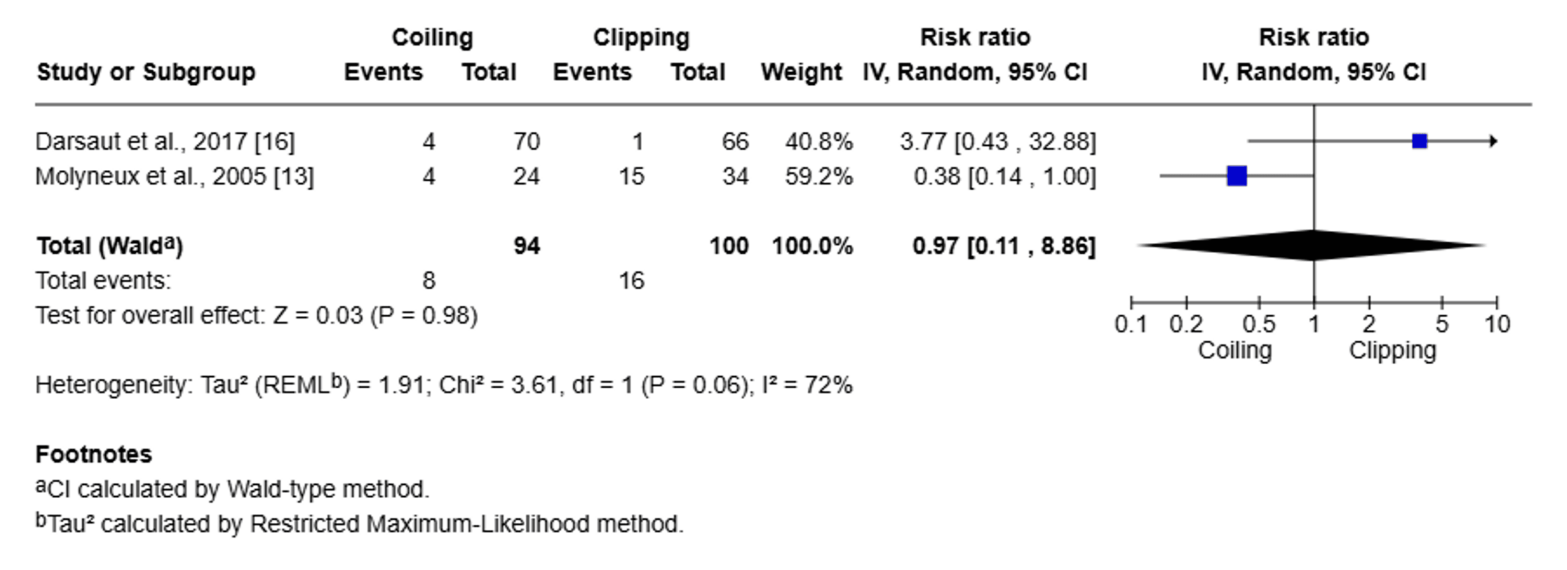
Forest plot showing the subgroup analysis of clipping and coiling for posterior circulation aneurysms

Subgroup analysis was done for posterior circulation aneurysms comparing clipping and coiling (Figure 9).

Two studies included 194 patients with posterior circulation aneurysm, and the pooled RR was 0.97 (95% CI: 0.11-8.86; Z = 0.03; P = 0.98), which shows no significant difference between coiling and clipping. While heterogeneity was high (I^2^ = 72%), that indicates variability between studies and limited reliability of the combined estimate. As the sample size is small with a wide confidence interval, therefore, no definitive conclusion can be drawn regarding the superiority of either treatment approach for posterior circulation aneurysms.

Result of bias analysis 

Publication Bias

**Figure 10:**
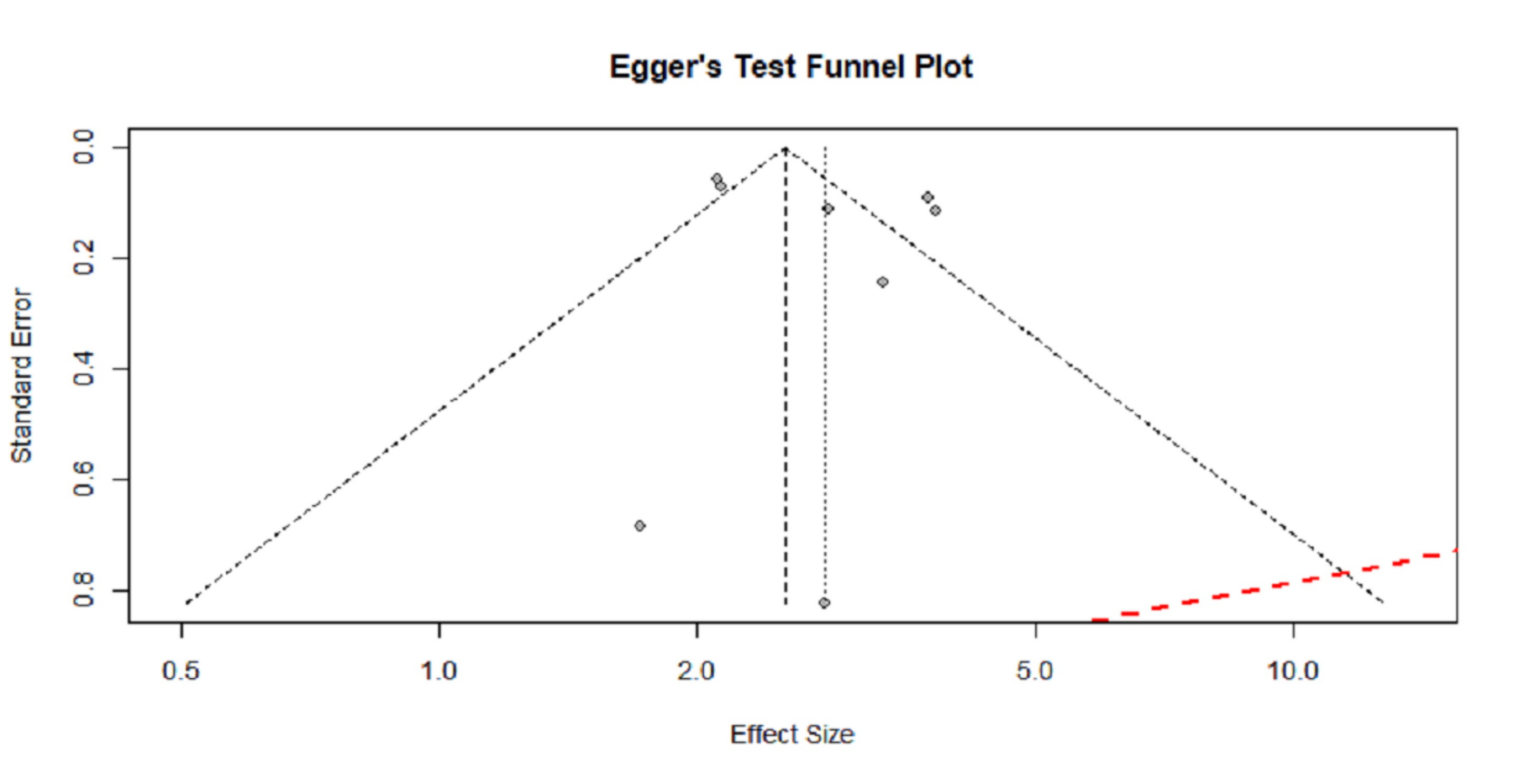
Egger's test funnel plot showing the publication bias of the eight included studies

Egger's test funnel plot was generated to determine the presence of publication bias, as shown in Figure 10. The study showed that there is asymmetry, with clustering of studies on the right side of the funnel and relative paucity on the left side, indicating the possibility of small study effects or publication bias (P = 0.3843). Egger's regression line has deviated from the vertical, further highlighting asymmetry. In addition, a trim-and-fill funnel plot was utilized to adjust publication bias. However, the method did not impute any missing studies, and the adjusted pooled effect remained largely unchanged, suggesting that although visual asymmetry was present, the statistical evidence for significant publication bias may be limited.

Discussion

Interpretation of Key Findings

This is the first meta-analysis to synthesize data on the impact of aneurysm locations and determine the optimal management strategy. Our meta-analysis showed that coiling has proven to be effective in terms of overall mortality reduction and has trended toward better functional outcomes at 1-5 years of follow-up, as described in other studies [20,21]. Our study findings also provided evidence that location-specific differences, such as clipping, tended to be more beneficial for MCA aneurysms. Coiling showed a non-significant benefit for AComA and was highly associated with reduced poor outcomes for ICA aneurysm. There is no statistically significant difference in the posterior circulation.

Comparison With Previous Studies

These findings are consistent with previous literature, such as the ISAT trials, which showed better short-term outcomes in AComA aneurysms and have been described in other studies as well. Our location-based analysis showed that clipping is a better modality for MCA aneurysms, which is consistent with the same evidence from trials from Ryttlefors et al. (ISAT-2) [19] and CURES trials for ruptured and unruptured MCA aneurysms and other literature [9,22]. For example, in a study, microsurgical clipping was performed on 120 MCA aneurysms, and endovascular clipping was performed on 40 aneurysms. It showed that technical treatment success was higher for clipping than for endovascular treatment for MCA aneurysms and the favorable outcome was 99.2% for clipping and 95% for coiling at the six-month follow-up for MCA aneurysms, suggesting clipping to be the standard treatment approach for MCA aneurysms [23].

It has also been shown in our results that coiling has a sufficient impact on ICA aneurysm location in terms of improved outcome, which is highly correlated with findings of both the Barrow Ruptured Aneurysm Trial (BRAT) and long-term ISAT trials. A study of 138 patients explained that coiling had shown fewer complications for ICA aneurysms than anterior cerebral artery aneurysms, with a 99.9% Raymond-Roy occlusion class I or II immediately and a 96.2% occlusion rate at the 12-month follow-up [24].

It was also observed for posterior circulation aneurysm in a retrospective cohort study conducted for the efficacy and effectiveness of EVT for posterior circulation aneurysm in 49 patients. While the result of the study revealed that 94% of the patients had successful occlusion of the aneurysm at 6-12 months of follow-up, 89.3% of the patients showed a one-year overall survival rate, emphasizing the effectiveness and safety of EVT for posterior circulation aneurysms [25].

Clinical implications

These findings suggest that choosing treatment based on the location of the aneurysm is important, indicating that coiling might be better for ICA aneurysms and possibly for AComA aneurysms if the anatomy allows. Clipping has been a perfect treatment option for MCA aneurysms because of favorable surgical access and long-term durability.

Heterogeneity and strength of evidence

Moderate heterogeneity was seen in the overall mortality meta-analysis (I^2^ = 48%), and low heterogeneity was observed in the functional independence outcome (I^2^ = 40%). Heterogeneity for the subgroup analysis of aneurysm locations is low for AComA and ICA aneurysms. In contrast, heterogeneity for MCA subgroups is substantial (I^2^ = 76%). These variations could be due to aneurysm morphology, surgical techniques, and surgical expertise across different studies. Despite these limitations, the overall direction of effect remained consistent in favoring coiling for most aneurysms.

Strengths and limitations

It is the first meta-analysis that systematically evaluated the aneurysm location as a predictor. This meta-analysis included subgroup analysis, which many meta-analyses overlook, and it is based on high-quality RCTs with large sample sizes (over 8000), with the use of both quantitative and qualitative analyses to synthesize findings. Despite these strengths, it has some limitations, for example, the subgroup analysis is limited by sample size, especially for posterior circulation aneurysms, and all the studies did not provide location-specific outcome data. There are a limited number of RCTs having significant publication bias in this meta-analysis.

## Conclusions

Coiling is associated with significantly lower mortality and improved functional outcomes compared to clipping in the overall treatment of ruptured intracranial aneurysms. Subgroup analysis suggests that treatment decisions should be tailored by aneurysm location, with clipping favored for MCA and coiling showing a trend toward benefit in AComA and a significant advantage in ICA aneurysms, while no clear superiority was observed in posterior circulation aneurysms. These findings support a selective, anatomy-driven approach to aneurysm management. Despite these findings, it is also being emphasized that the selection between clipping and cooling should not be looked at as a uniform decision. There are other factors as well, such as age, aneurysm morphology and size, and surgical accessibility and expertise. All of them play a crucial role in determining optimal intervention. Coiling provides the advantages of lower perioperative morbidity and faster recovery. But there are some disadvantages associated with coiling, such as durability and the likelihood of retreatment. In contrast, clipping is advantageous in providing a more definitive cure in a suitable anatomical location, but it is associated with immediate increased surgical complications. Therefore, incorporation of multidisciplinary expertise such as neurosurgery, interventional neuroradiology, and critical care is important to tailor strategies and optimize outcomes.
